# Clinical and genomic predictors of sipuleucel-T outcomes in men with metastatic androgen pathway modulator resistant prostate cancer

**DOI:** 10.21203/rs.3.rs-10042173/v1

**Published:** 2026-07-01

**Authors:** Andrew Armstrong, Tara Seibert, Jane McKenzie, Lauren Howard, Bilal Ashraf, Jasmine Lu, Kallie White, Daniel George, Jeffrey Shevach, Joseph Park, Dillon Cockrell, Kristen Davis, Michael Harrison, Christopher Hoimes, Jordan Infield, Matthew Labriola, Hannah McManus, Sundhar Ramalingam

**Affiliations:** Duke University Medical Center; Duke Cancer Institute Center for Prostate and Urologic Cancers, Duke University; Duke Cancer Institute Center for Prostate and Urologic Cancers, Duke University; Duke University School of Medicine; Duke University Medical Center; Duke Cancer Institute Center for Prostate and Urologic Cancers, Duke University; Duke University Medical Center; Duke Cancer Institute, Duke University School of Medicine; Duke University; Medical Oncology and Duke Cancer Institute, Duke University, Durham, NC and Durham Veterans Affairs Medical Center, Durham, NC; Duke University; Duke Cancer Institute Center for Prostate and Urologic Cancers, Duke University; Duke Cancer Institute Center for Prostate and Urologic Cancers; Case Comprehensive Cancer Center, University Hospitals Seidman Cancer Center, Case Western Reserve University, Cleveland, OH, USA, and Duke University School of Medicine; Duke University; Duke University Medical Center; Duke University

## Abstract

**BACKGROUND:**

Survival outcomes following sipuleucel-T in men with metastatic androgen pathway modulator resistant prostate cancer (mAPMR) are variable. Genomic alterations influencing immune response and tumor proliferation may explain outcome heterogeneity. We sought to identify clinical and genomic correlates of survival following sipuleucel-T.

**METHODS:**

We conducted a single-center retrospective study of men with mAPMR and accessible tumor genomic data who received sipuleucel-T at the Duke Cancer Institute (2014–2024). The primary objective was to associate clinical factors and somatic genomic alterations with overall survival (OS) using multivariable Cox models.

**RESULTS:**

Among 429 men treated with sipuleucel-T, 185 (43%) had molecular data (tumor tissue 43%, cfDNA 57%). Median age was 70; most were ECOG 0 (60%) and White (85%) or Black (14%). Frequent alterations included TP53 (48%), AR amplification (27%), PTEN loss (20%), and MYC gain (10%). Median OS and progression-free survival (PFS) were 44 and 3.6 months, respectively. MYC gain was the strongest independent genomic predictor of poorer OS. On multivariable analysis, predictors of poorer OS included ≥ 10 bone metastases (HR 42.4, 95% CI 10.3–175, p < 0.001), MYC gain (HR 7.96, 95% CI 2.88–22.0, p < 0.001), older age (HR 1.05, 95% CI 1.01–1.09, p = 0.017), TMPRSS2 wild type vs. mutation (HR_mutation_ 0.35, 95% CI 0.16–0.78, p = 0.011), higher alkaline phosphatase (HR 1.11 per 10 IU/L, 95% CI 1.04–1.18, p < 0.001), and higher ANC (HR 1.30, 95% CI 1.06–1.58, p = 0.011).

**CONCLUSIONS:**

MYC gain is strongly associated with poorer OS following sipuleucel-T. Aggressive tumor biology, immune evasion, and advanced disease state may underlie these inferior outcomes; alternative therapies should be considered in MYC-amplified mAPMR. Multicenter validation is planned to further enhance patient selection for sipuleucel-T.

## Introduction

Advances in cancer therapeutics have transformed the treatment paradigm for metastatic androgen pathway modulator resistant prostate cancer (mAPMR, the new preferred term for castration-resistant prostate cancer) (1). Multiple life-prolonging treatments now carry FDA approval, including the autologous cellular vaccine sipuleucel-T (2). Sipuleucel-T stimulates a T-cell-mediated immune response against prostate acid phosphatase, an antigen widely expressed in prostate cancer. In phase III trials, sipuleucel-T significantly improved overall survival (OS) compared to placebo and was approved in asymptomatic/minimally symptomatic mAPMR (2–4). However, survival outcomes vary substantially, and the mechanism(s) driving these differential responses remain poorly understood. Identifying clinical and genomic features predictive of survival may optimize patient selection for sipuleucel-T in mAPMR.

Prior studies have highlighted multiple clinical predictors of OS following sipuleucel-T, including prostate-specific antigen (PSA), lactate dehydrogenase (LDH), alkaline phosphatase (ALP), hemoglobin, age, race/ethnicity, Eastern Cooperative Oncology Group (ECOG) performance status, time since diagnosis, lymph node only metastases, prior abiraterone/enzalutamide, and prior docetaxel/cabazitaxel (5–8). Prolonged OS has been reported among patients with features of lower disease burden, including better PS, lower baseline PSA quartile, and favorable PSA kinetics (5–9). Proposed explanations include less immunosuppression with lower disease burden conferring a more robust immunotherapy response, earlier treatment initiation allowing time for delayed onset of action, and variability in immunologic profiles (5, 7).

Tumor genomics and their influence on immune response or tumor growth kinetics may also contribute to differential outcomes. Molecular features increasing tumor immunogenicity may confer an enhanced response to sipuleucel-T, whereas alterations inducing immune evasion or rapid tumor proliferation may predict poorer outcomes (10, 11). Certain alterations, such as RB1 loss, MYC gain, TP53 mutations, and PTEN loss, have been linked to inferior outcomes across mAPMR therapies (12–17). In particular, PTEN loss may portend poorer survival with sipuleucel-T through T cell exclusion and an immunosuppressive effect on the tumor microenvironment (18, 19). Conversely, CDK12 loss is associated with increased neoantigen burden and immune infiltration, potentially enhancing response to immunotherapy (20). Black men have a higher incidence of CDK12 mutations and MSI-high disease, and lower rates of PTEN alterations compared to White men, which may partly explain previously reported improved outcomes with sipuleucel-T for Black men (21, 22).

We thus hypothesized that genomic alterations related to immunomodulation and tumor growth kinetics would correlate with differential OS following sipuleucel-T.

## Materials/Subjects and Methods

### Study Design

We performed a single-center retrospective cohort analysis derived from the electronic health record and Duke Molecular Registry of Tumors. Eligible patients were adults with mAPMR who received ≥1 sipuleucel-T infusion at Duke Cancer Institute between 2014–2024 and had accessible somatic genomic testing. Patients lacking ≥6-month follow-up data were excluded to ensure adequate time had elapsed to assess the intervention. This study was approved by the Duke University Health System institutional review board (Pro00116941) with waiver of informed consent.

### Study Objectives & Endpoints

The primary objective was to evaluate the associations between specific somatic genomic alterations and OS. We hypothesized that patients with CDK12 mutations, PTEN wild type tumors, and/or MSI-high disease would have improved outcomes with sipuleucel-T, while those with PTEN loss/mutations, TP53/RB1 loss/mutations, and/or MYC amplification would have inferior outcomes. The primary endpoint was OS, defined as time from first sipuleucel-T infusion to death from any cause. Secondary endpoints included real-world progression-free survival (PFS), time to next therapy, and PSA response. Real-world PFS was defined as time from first sipuleucel-T infusion to clinical, PSA, or radiographic progression, treatment change, or death. PSA progression was defined as a ≥25% increase and ≥2 ng/mL rise from the baseline or nadir PSA (23). Time to next therapy was defined as time from first sipuleucel-T infusion to initiation of subsequent cancer therapy. Percent change in PSA was calculated from baseline PSA, which was defined as the most recent value prior to infusion, and nadir PSA post-infusion. Nadir PSA values occurred any time following sipuleucel-T and prior to subsequent lines of therapy.

### Statistical Analysis

Baseline characteristics and genomic alterations were summarized using medians, interquartile ranges, and range for continuous variables, and frequencies and percentages for categorical variables. Analyses involving genomic data were restricted to patients with next-generation sequencing (NGS) performed contemporary to sipuleucel-T – defined as prior to or within 6 months of sipuleucel-T initiation. Genetic alterations were compared by race, receipt of intercurrent therapy, and by MYC amplification. Differences were tested using Wilcoxon rank sum test for continuous comparisons (i.e. number of alterations) or chi-squared or Fisher’s exact test for categorical variables.

Kaplan-Meier estimates were used to derive median OS and 36-month survival across relevant patient and disease characteristics. Univariable Cox regression models were used to evaluate associations between demographic, disease, and genetic factors and OS. Elastic net was used for variable selection and ranking. Selected variables were then included in an unpenalized multivariable Cox proportional hazards model for estimation of effect sizes. Hazard ratios (HRs) and 95% confidence intervals (CIs) from the multivariable model were shown in a forest plot. To assess PFS and time to next therapy, Kaplan-Meier curves and Cox models were used as outlined for the primary objective. Percent change in PSA from baseline was calculated by disease factors. Statistical analyses were performed using R (R Foundation for Statistical Computing, Vienna, Austria). All tests were two-sided, and p values < 0.05 were considered statistically significant.

## Results

### Baseline Characteristics

From 2014–2024, we identified 429 patients with mAPMR who received ≥1 sipuleucel-T infusion (Supplementary Fig. 1). Of these, 185 patients (43.1%) had genomic data available and 84 patients (45%) had contemporary NGS. Baseline demographic and clinical characteristics are summarized in Table 1. Median age was 70 (range 48–95); most men were ECOG 0 or Karnofsky Performance Scale 90–100 (60%) and were White (84.9%) or Black (14.1%). Median PSA was 2 ng/mL (range 0–472 ng/mL). Pattern of spread included lymph node only (16%), bone only (49%), bone plus lymph node (22%), and visceral metastases (3.8%). Prior therapies included radical prostatectomy or radiation therapy (66%), androgen deprivation therapy (ADT) (99.5%), androgen receptor pathway inhibitor (ARPI) (20%), and chemotherapy (27%). Baseline characteristics were similar between patients with and without contemporary NGS, and between those receiving intercurrent therapy versus sipuleucel-T monotherapy, with the exception that high volume disease was more common in the intercurrent therapy group. High volume was defined by the CHAARTED criteria (presence of visceral metastases or ≥ 4 bone lesions with ≥ 1 extra-axial lesion) (24).

### Genomic Data

Among 185 patients with genomic testing, specimen sources included cfDNA in 106 patients (57%) and tumor tissue ± cfDNA in 79 patients (43%). NGS was performed using FoundationOne and Guardant 360 assays. Genomic alterations were detected in 77% of cfDNA samples, including 68% with contemporary NGS and 81% with testing after sipuleucel-T. In tumor tissue ± cfDNA samples, alterations were detected in 97.5%, including 96.4% with contemporary NGS and 100% with testing after sipuleucel-T. Contemporary NGS results are summarized in Supplementary Table 1.

### Genomic Data: Subgroup analyses

In patients with contemporary NGS, the only statistically significant difference in alteration frequencies by race was in TMPRSS2, occurring in 0% of Black patients vs. 32% of non-Black patients (p = 0.031).

Patients with MYC amplification (n = 10) compared to those without (n = 74) had a higher median number of alterations (4 vs. 3, p < 0.001), a greater proportion with ≥3 alterations (p = 0.004), and higher frequencies of TP53 mutations (80% vs. 38%, p = 0.016) and AR amplification (30% vs. 5.4%, p = 0.034).

### Overall Survival

**Figure 1:**
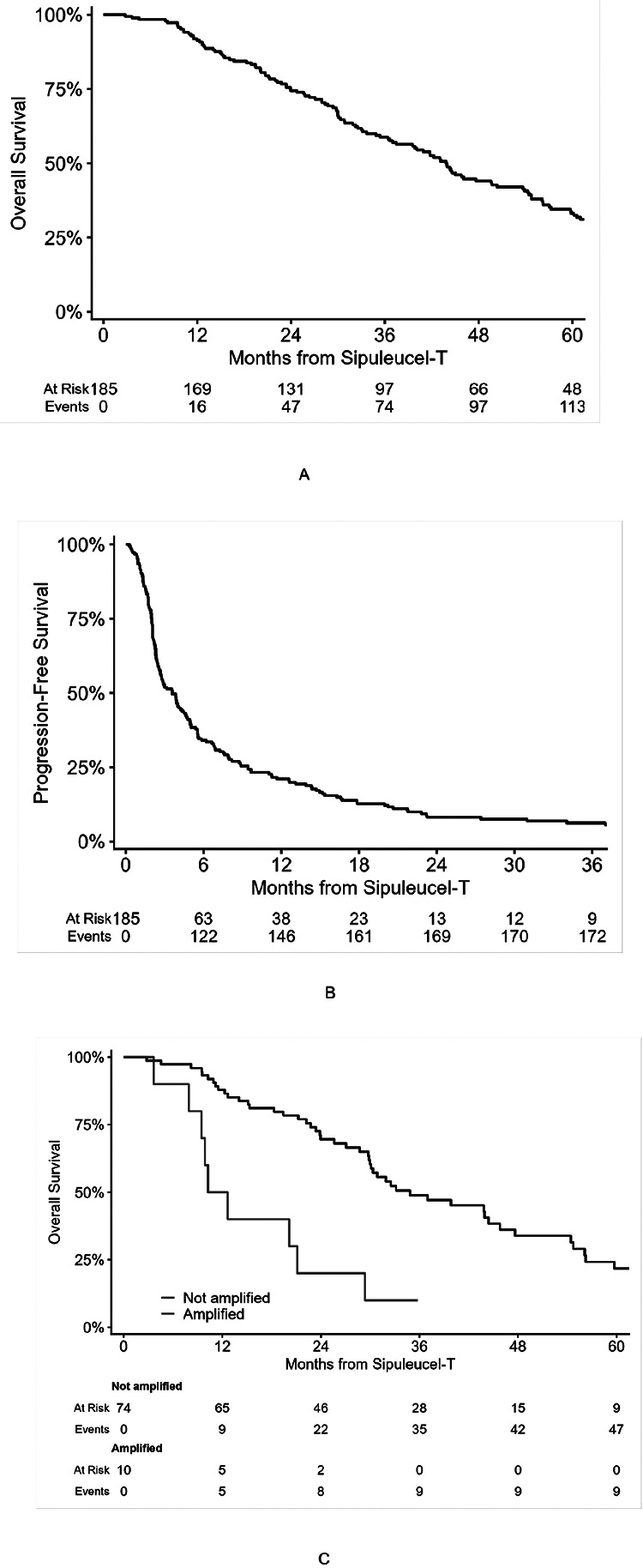
OS and PFS KM curves for total cohort (Figures 1A and 1B) and OS by MYC status (Figures 1C). The Kaplan-Meier curves stratified by genetic mutations are restricted to patients with contemporary genetic testing.

Median follow-up was 100 months. Median OS was 44 months (95% CI 37–54) in the overall cohort and 32 months (95% CI 29–44) among patients with contemporary NGS. Survival curves are shown in Fig. 1. Among patients with contemporary NGS, 90% of those with MYC gain had died, compared to 47% of patients without MYC alterations. Patients with MYC gain experienced shorter median OS than those without (11 vs. 35 months) and lower 3-year survival rates (10% vs. 49%). Receipt of intercurrent therapy was also associated with reduced OS (37 vs. 50 months). Median OS was similar between Black and non-Black patients (45 vs. 43 months) and between patients with and without PTEN loss (30 vs. 32 months). Survival data by genomic information is shown in Table 2.

In univariable analyses, poorer OS was associated with older age (HR 1.04, 95% CI 1.00–1.07, p = 0.030), receipt of intercurrent radium-223 (HR 4.74, 95% CI 1.11–20.2, p = 0.035), PSA in the highest quartile (HR 2.35, 95% CI 1.13–4.89, p = 0.022), > 10 bone metastases (HR 8.92, 95% CI 3.01–26.2, p < 0.001), lower hemoglobin (HR 0.77, 95% CI 0.64–0.93, p = 0.007), higher LDH (HR 1.00, 95% CI 1.00–1.00, p = 0.030), lower albumin (HR 0.19, 95% CI 0.07–0.53, p = 0.001), and higher ALP (HR 1.09 per 10 IU/L, 95% CI 1.04–1.13, p = 0.049). Of the genomic alterations, mAPMR patients with AR amplification (HR 5.26, 95% CI 2.30–12.1, p < 0.001), MYC gain (HR 4.25, 95% CI 2.01–8.99, p < 0.001), and TP53 (HR 2.36, 95% CI 1.39–3.99, p = 0.001) had significantly poorer survival, while those without detectable alterations (HR≥ 1 alteration 0.22, 95% CI 0.05–0.89, p = 0.034) had improved survival. PTEN loss was not associated with OS (HR 1.12, 95% CI 0.62–2.03, p = 0.7).

**Figure 2:**
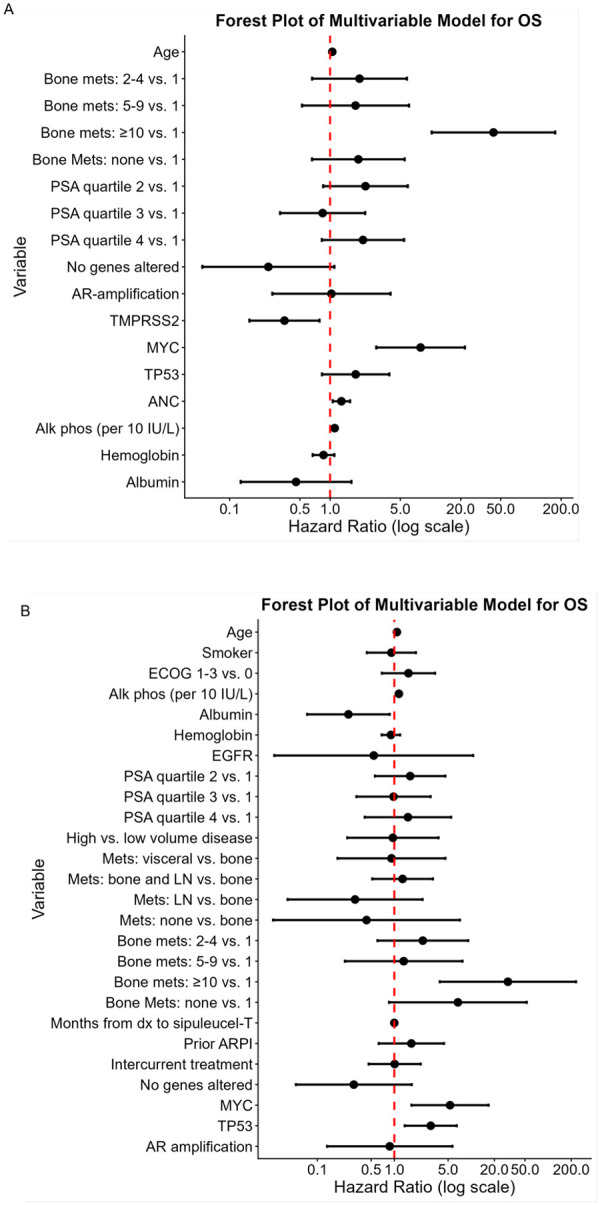
Forest plot of multivariable model for OS (Figure 2A) and PFS (Figure 2B); variables were selected using elastic net and hazard ratios were estimated from a multivariable Cox model.

Elastic net modeling identified ≥ 10 bone lesions and MYC gain as the strongest predictors of shorter OS. An unpenalized multivariable Cox model confirmed the following independent predictors of worse OS: ≥10 bone lesions (HR 42.4, 95% CI 10.3–175, p < 0.001), MYC gain (HR 7.96, 95% CI 2.88–22.0, p < 0.001), older age (HR 1.05, 95% CI 1.01–1.09, p = 0.017), TMPRSS2 wild type vs. mutation (HR_mutation_ 0.35, 95% CI 0.16–0.78, p = 0.011), higher ALP (HR 1.11 per 10 IU/L, 95% CI 1.04–1.18, p < 0.001), and higher ANC (HR 1.30, 95% CI 1.06–1.58, p = 0.011) (Fig. 2).

### PFS

**Figure 3:**
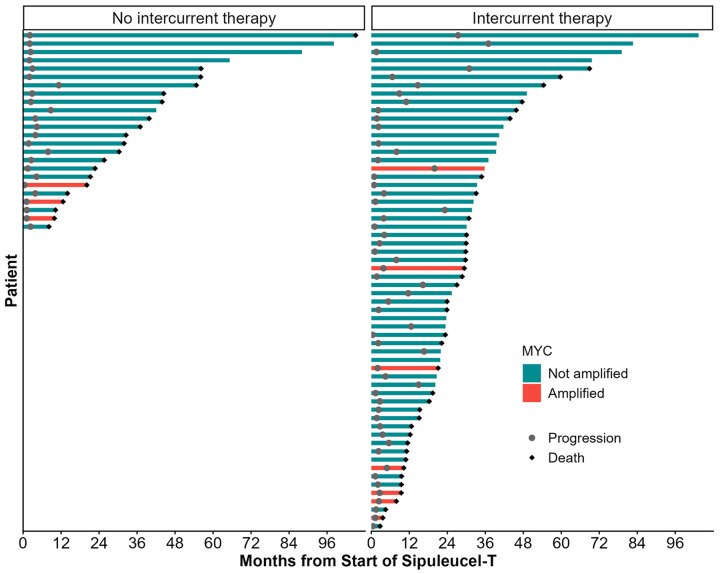
Swimmer Plot showing time to progression and death among patients who received sipuleucel-T monotherapy (Figure 3A) vs. intercurrent therapy (Figure 3B). Patients with MYC amplification are annotated.

Median PFS for the overall cohort was 3.6 months (95% CI 2.7–4.6). Among patients with contemporary NGS, median PFS was shorter for those with MYC gain than those without (2.2 vs. 2.8 months). Intercurrent therapy was associated with longer PFS (5.0 vs. 2.5 months). PFS and OS for sipuleucel-T monotherapy vs. intercurrent therapy with annotation of MYC gain status are shown in Fig. 3. Median PFS was 2.6 months for Black patients vs. 3.8 months for non-Black patients.

### Predictors of PFS

In univariable analyses, several clinical and genomic factors were significantly associated with shorter PFS, including baseline PSA quartile 4 (HR 2.20, 95% CI 1.16–4.17, p = 0.016), higher NLR (HR 1.12, 95% CI 1.05–1.20, p < 0.001), higher LDH (HR 1.00, 95% CI 1.00–1.00, p = 0.004), higher ALP (HR 1.00 per 10 IU/L, 95% CI 1.00–1.01, p = 0.038), lower albumin (HR 0.45, 95% 0.21–0.98, p = 0.043), AR resistance (HR 2.48, 95% CI 1.17–5.28, p = 0.018), BRCA2 mutation (HR 2.34, 95% CI 1.21–4.53, p = 0.011), and ≥3 genomic alterations (HR 1.64, 95% CI 1.04–2.59, p = 0.033). Receipt of intercurrent therapy (HR 0.52, 95% CI 0.32–0.87, p = 0.013) was associated with longer PFS, while receiving radium-223 vs. ARPI (HR 20.7, 95% CI 4.37–98.0, p < 0.001) was associated with shorter PFS.

Elastic net modeling identified number of sipuleucel-T infusions as the strongest predictor of PFS; however, this association was not retained in the unpenalized multivariable Cox model. The unpenalized multivariable Cox model confirmed the following independent predictors of PFS: BRCA2 mutation (HR 4.50, 95% CI 1.80–11.2, p = 0.001), receipt of intercurrent treatment (HR 0.38, 95% CI 0.18–0.80, p = 0.011), AR resistance (HR 3.85, 95% CI 1.29–11.5, p = 0.015), higher ALP (HR 1.07 per 10 IU/L, 95% CI 1.02–1.13, p = 0.011), TP53 alteration (HR 1.99, 95% CI 1.04–3.82, p = 0.039), and higher NLR (HR 1.21, 95% CI 1.09–1.34, p < 0.001).

### PSA outcomes and time to next therapy

The median time to PSA progression was 3.9 months (95% CI 2.6–5.0) for patients with contemporary NGS. Patients with MYC gain progressed more rapidly than those without (2.2 vs. 3.9 months), as did Black patients compared with non-Black patients (3.3 vs. 3.9 months). Intercurrent therapy was associated with longer PSA PFS (4.6 vs. 2.4 months).

Median PSA change following sipuleucel-T was + 38%. Median PSA increase was greater among patients with MYC gain vs those without (+ 111% vs. +26%) and Black patients vs non-Black patients (+ 77% vs. +26%), and smaller for patients who received intercurrent therapy vs monotherapy (+ 13% vs. +65%).

For the overall cohort and subgroup with contemporary NGS, the median time to next therapy was 7.1 months (95% CI 5.7–9.1) and 4.9 months (95% CI 3.8–8.4), respectively. Patients with MYC gain had a shorter time to next therapy than those without (3.5 vs 5.3 months). Patients receiving intercurrent therapy had a longer time to next therapy compared with monotherapy (10 vs. 4.2 months). Black patients had similar time to next therapy compared to non-Black patients (7.0 vs. 7.1 months).

## Discussion

In this retrospective, single-center study of men with mAPMR, we identified several novel clinical and genomic predictors of differential outcomes with sipuleucel-T. Chiefly, MYC gain was associated with shorter PFS, greater PSA rises post-treatment, shorter time to next therapy, and worse OS. Additional predictors of worse OS included higher number of bone metastases, higher ANC, older age, higher ALP, and TMPRSS2 wild type status.

The association between MYC gain and poor outcomes is likely multifactorial. First, MYC gain is linked to aggressive, proliferative disease across the spectrum of prostate cancer. In vitro, MYC gain disrupts AR transcriptional programming and accelerates development of mAPMR (25). MYC or MYCN gain also drives lineage plasticity and development of neuroendocrine prostate cancer (26). Clinically, MYC gain is associated with higher Gleason score and increased post-prostatectomy recurrence (27). In mAPMR, it is associated with aggressive variant prostate cancer (AVPC) features, such as liver metastases (28).

Second, MYC plays a central role in immune evasion, likely conferring resistance to sipuleucel-T. MYC promotes cytokine expression, including IL-13 and CCL2, which induce immunosuppressive macrophages, thereby inhibiting recruitment of B cells, natural killer cells, and CD4+/CD8 + T cells (29, 30). Further, MYC upregulates checkpoint proteins, including PD-L1, causing immune evasion via T-cell exhaustion (31, 32). This proliferative, immune-evasive phenotype is therefore less likely to respond to sipuleucel-T. Our findings suggest alternative treatments should be considered in men with MYC-amplified mAPMR when feasible.

The clinical factors associated with poorer outcomes in this study are consistent with existing literature. Sipuleucel-T was approved for asymptomatic/minimally symptomatic mAPMR based on the IMPACT trial (3). Patients with extensive bone metastases or elevated ALP levels are more likely to be symptomatic and may not have met eligibility criteria in the original study. Similar results were seen in the PROCEED registry, the largest real-world data study of sipuleucel-T (8). Together, these findings reinforce the importance of careful patient selection for sipuleucel-T.

Although race predicted PFS in our study, it was not associated with OS, contrary to the aforementioned PROCEED and IMPACT studies, where Black patients demonstrated improved OS (3, 8). Our smaller sample size likely limited power to detect genomic differences between Black and non-Black patients. We observed lower rates of TMPRSS2 mutations and PTEN loss among Black patients, consistent with prior reports (33). However, we did not observe other reported genomic differences, including the immunogenic SPOP or CDK12 mutations, limiting interpretation of racial outcome disparities (34, 35). Additionally, the limited catchment area of a single-center study may have resulted in ancestral overlap and similar genomic profiles regardless of self-identified race.

Notably, 43% of men treated with sipuleucel-T in our cohort received NGS testing, exceeding real-world ranges of 19–27% (36). These findings further support expanded genetic testing in metastatic prostate cancer, particularly mAPMR. This is corroborated by our observation that MYC gain predicted poorer outcomes with sipuleucel-T. This is especially relevant in AVPC, an aggressive subtype of mAPMR with particularly poor outcomes (37). AVPC is molecularly defined by alterations in at least two of TP53, RB1, and PTEN (38). As treatment options for mAPMR continue to evolve, clinicians will increasingly need more patient-centered data, such as somatic tissue NGS, to individualize treatment selection, improve prognostication, and guide discussions.

This study has several limitations. Its retrospective design introduces potential selection bias in patients offered NGS. Second, the single-center setting limits sample size. Nonetheless, to our knowledge, this is the largest study evaluating genomic correlates of sipuleucel-T outcomes in men with mAPMR.

## Supplementary Material

Supplementary Files

This is a list of supplementary files associated with this preprint. Click to download.

- table2.xlsx
- table1.xlsx
- supplementarytable1.xlsx

Tables 1 and 2 are available in the Supplementary Files section.

## Data Availability

We can provide the datasets to interested third parties only upon request and cannot submit human subjects data for public use without permission. We can make a de-identified dataset available upon request and with appropriate IRB approvals in place.
